# Ultrasonographic changes in quadriceps femoris thickness in women with normal pregnancy and women on bed rest for threatened preterm labor

**DOI:** 10.1038/s41598-022-22467-8

**Published:** 2022-10-19

**Authors:** Yohei Takahashi, Takashi Kaji, Toshiyuki Yasui, Atsuko Yoshida, Naoto Yonetani, Naoto Suzue, Shinsuke Katoh, Kazuhisa Maeda, Koichi Sairyo, Minoru Irahara, Takeshi Iwasa

**Affiliations:** 1 Department of Obstetrics and Gynecology, Institute of Biomedical Sciences, Tokushima University Graduate School, Tokushima, 770-8503 Japan; 2 Department of Reproductive and Menopausal Medicine, Institute of Biomedical Sciences, Tokushima University Graduate School, Tokushima, Japan; 3 Department of Orthopedic Surgery, Tokushima Red Cross Hospital, Komatsushima, Japan; 4 Department of Rehabilitation Medicine, Red Cross Tokushima Hinomine Rehabilitation Center for People with Disabilities, Komatsushima, Japan; 5 Department of Obstetrics and Gynecology, Shikoku Medical Center for Children and Adults, Zentsuji, Japan; 6 Department of Orthopedics, Institute of Biomedical Sciences, Tokushima University Graduate School, Tokushima, Japan

**Keywords:** Medical imaging, Preterm birth, Muscle

## Abstract

This study aimed to evaluate the changes in quadriceps femoris muscle thickness during the pregnancy and postpartum periods and to elucidate the effect of bed rest for threatened preterm labor on muscle thickness. In 26 women with normal pregnancy, quadriceps femoris thickness was measured at 11–13, 26, 30, and 35 weeks’ gestation, and at 3–5 days and 1 month postpartum using ultrasonography. In 15 pregnant women treated with bed rest for threatened premature labor, quadriceps femoris thickness was measured at 30 and 35 weeks’ gestation and postpartum. In women with normal pregnancy, quadriceps femoris thickness increased, peaking at 35 weeks’ gestation, followed by a postpartum decrease. In women on bed rest, quadriceps femoris thickness showed no significant change during the pregnancy and postpartum periods, and the muscle was significantly thinner at 35 weeks’ gestation than that in women with normal pregnancy. In conclusion, a significant increase in quadriceps femoris muscle thickness during normal pregnancy was found using ultrasonography. Meanwhile, in pregnant women on bed rest treatment, the quadriceps femoris was significantly thinner in the late third trimester than that in normal pregnant women. Prolonged bed rest can affect normal changes in the quadriceps femoris muscle thickness during the pregnancy and postpartum periods.

## Introduction

Loss of muscle volume, such as in locomotive syndrome or sarcopenia, has been reported to be related to various diseases including diabetes mellitus, hypertension, and cardiovascular disease, suggesting that it is important to maintain the muscle volume^1–3^. Assessment of muscle volume is performed to detect muscle wasting in individuals with various diseases and conditions, such as cancer, heart failure, and immobilization because muscle wasting is associated with a poor prognosis^4–6^. Ultrasonography has been used to measure muscle thickness to assess the muscle volume because of its convenience, non-invasiveness, and the correlation of the ultrasonographic findings with the muscle volume calculated by magnetic resonance imaging (MRI)^7–9^. Previous reports used the measurement of quadriceps femoris muscle thickness to estimate the muscle volume and monitor muscle wasting in men and non-pregnant women during bed rest^7–9^. Bed rest decreased the ultrasonographic muscle thickness of the quadriceps femoris, indicating a loss of the quadriceps femoris muscle volume^7–9^. The quadriceps femoris muscle, which is an antigravity muscle, plays an important role in extending the knee joint and maintaining the standing position. Loss of quadriceps femoris muscle volume hampers the ability to walk and maintain the balance^10,11^. In sarcopenia, quadriceps femoris muscle volume was found to be lower than that of other skeletal muscles^12^.

Preterm labor, defined as labor occurring before 37 weeks of pregnancy, accounts for 5–18% of all deliveries^13^. Bed rest is widely prescribed to pregnant women to prevent preterm labor despite the potential maternal risks and lack of proven efficacy^14^. We previously reported that long-term bed rest for threatened premature labor (TPL) affected the maternal bone metabolism during pregnancy and the postpartum period^15,16^. A study reported that bed rest treatment during pregnancy impaired reoxygenation of the gastrocnemius muscle, indicating that immobilization during pregnancy affects maternal muscle metabolism^17^. However, the change in the muscle volume during pregnancy and the postpartum period in women with normal pregnancy, and the effect of bed rest treatment on the muscle volume, have not been reported to date.

Therefore, the aims of this study were to evaluate the changes in the muscle thickness of the quadriceps femoris during pregnancy and the postpartum period as a longitudinal study, and to elucidate the effect of bed rest treatment for TPL on the muscle thickness during pregnancy and the postpartum period using ultrasonography.

## Results

### Baseline characteristics of women with normal pregnancy

The baseline characteristics are summarized in Table 1. The average duration of pregnancy was 38.9 ± 1.0 weeks. The mean body weight before pregnancy was 49.7 ± 5.5 kg, and the mean body weight gain during pregnancy was 11.1 ± 3.2 kg. The body weight at 1 month postpartum was 53.5 ± 6.1 kg, and the body weight loss during the postpartum period (absolute value) was 7.4 ± 2.6 kg. The intake of calories and protein, and total physical activity at 30 weeks of pregnancy are shown in Table 1.

**Table 1:** Demographic characteristics in 26 women with normal pregnancy.

Variables	Subjects (n = 26)
Age at delivery (years)	31.8 ± 4.1
Nulliparity (%)	42
Body height (cm)	158.5 ± 4.2
Body weight before pregnancy (kg)	49.7 ± 5.5
BMI before pregnancy (kg/m^2^)	19.8 ± 2.1
Body weight gain during pregnancy (kg)	11.1 ± 3.2
Body weight at 1 month postpartum (kg)	53.5 ± 6.1
Intake of calories at 30 weeks of pregnancy (kcal/day)	1822.9 ± 465.5
Intake of protein at 30 weeks of pregnancy (g/day)	63.4 ± 17.4
Total physical activity at 30 weeks of pregnancy (MET minutes/week)	1942.5 (856.5 to 4254)
Weeks of pregnancy at delivery (weeks)	38.9 ± 1.0
Vaginal delivery (%)	92

Data are expressed as mean ± standard deviation, median (interquartile range), or n (%).

*BMI* body mass index, *METs* metabolic equivalents.

### Changes in the muscle thickness of the rectus femoris and vastus intermedius in women with normal pregnancy

**Figure 1:**
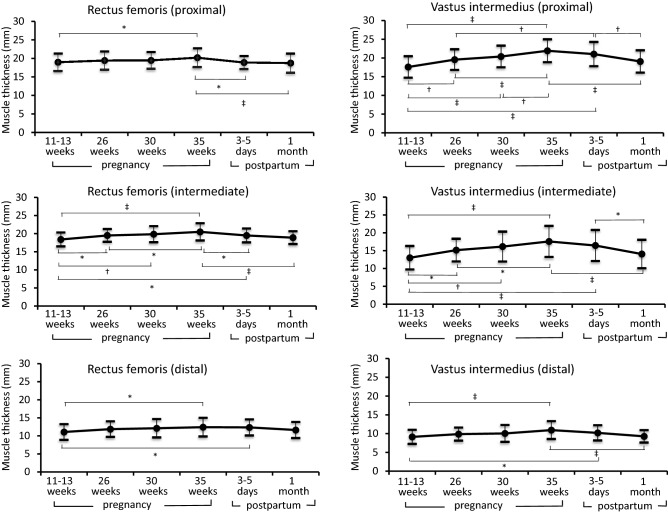
Changes in the muscle thickness in women with normal pregnancy. Changes in the muscle thickness of the rectus femoris (proximal, intermediate, and distal points) and vastus intermedius (proximal, intermediate, and distal points) in women with normal pregnancy are shown. Data are presented as mean ± standard deviation (*p < 0.05; ^†^p 0.01; ^‡^p < 0.001).

As shown in Fig. 1, the muscle thicknesses of all six points at 35 weeks of pregnancy were significantly greater than those at 11–13 weeks of pregnancy (proximal point of vastus intermedius: 21.9 ± 3.1 vs. 17.6 ± 2.9 mm, p < 0.001; intermediate point of vastus intermedius: 17.6 ± 4.4 vs. 13.0 ± 3.4 mm, p < 0.001; distal point of vastus intermedius: 10.9 ± 2.4 vs. 9.1 ± 1.9 mm, p < 0.001; proximal point of rectus femoris: 20.2 ± 2.6 vs. 18.9 ± 2.4 mm, p < 0.05; intermediate point of rectus femoris: 20.5 ± 2.4 vs. 18.4 ± 2.0 mm, p < 0.001; and distal point of rectus femoris: 12.4 ± 2.6 vs. 11.1 ± 2.2 mm, p < 0.05). In contrast, at 1 month postpartum, the muscle thicknesses of all six points except the distal point of the rectus femoris were significantly lower than those at 35 weeks (proximal point of vastus intermedius: 19.1 ± 3.0 vs. 21.9 ± 3.1 mm, p < 0.001; intermediate point of vastus intermedius: 14.0 ± 4.1 vs. 17.6 ± 4.4 mm, p < 0.001; distal point of vastus intermedius: 9.3 ± 1.7 vs. 10.9 ± 2.4 mm, p < 0.001; proximal point of rectus femoris: 18.7 ± 2.6 vs. 20.2 ± 2.6 mm, p < 0.001; intermediate point of rectus femoris: 18.9 ± 1.8 vs. 20.5 ± 2.4 mm, p < 0.001).

### Comparison of baseline characteristics between women with normal pregnancy and women on bed rest during pregnancy

The duration of pregnancy in women on bed rest for TPL was significantly shorter than that in women with normal pregnancy (37.8 ± 1.4 vs. 38.9 ± 1.0 weeks, p < 0.01). Among women on bed rest for TPL, the mean gestational weeks at admission was 23.7 ± 3.2 weeks. The mean gestational age at discharge was 36.1 ± 0.3 weeks. The mean duration of hospitalization (bed rest) was 86.3 ± 23.9 days. There were no significant differences between the two groups with regard to age. Regarding the maternal physical parameters, there were no significant differences between the two groups in body height, body weight before pregnancy, body mass index before pregnancy, and body weight at delivery, and at every ultrasonographic measurement time point from 11 to 13 weeks of pregnancy to 1 month postpartum. However, the gains in body weight from 11–13 to 26 weeks, from 11–13 to 30 weeks, and from 11–13 to 35 weeks of pregnancy in women on bed rest for TPL were significantly (p < 0.01) lower than those in women with normal pregnancy (26 weeks: 3.5 ± 1.7 vs. 5.4 ± 1.7 kg, 30 weeks: 4.5 ± 2.3 vs. 7.0 ± 2.1 kg, 35 weeks: 6.2 ± 2.9 vs. 8.7 ± 2.4 kg). There was no significant difference in the body weight at 1 month postpartum between the two groups. In contrast, the loss in body weight (absolute value) during the postpartum period in women on bed rest was significantly lower than that in women with normal pregnancy (4.8 ± 2.2 vs. 7.4 ± 2.6 kg, p < 0.01). The median and interquartile range (IQR) of total physical activities at 30 and 35 weeks of pregnancy in women on bed rest were significantly (p < 0.001 and p < 0.001, respectively) lower than those in women with normal pregnancy (30 weeks of pregnancy: 0 [0 to 0] vs. 1942.5 [856.5 to 4254.0], 35 weeks of pregnancy: 0 [0 to 0] vs. 1254 [881.3 to 2161.5] metabolic equivalents (METs)-min/week). However, there was no significant difference in the total physical activity at 1 month postpartum between women on bed rest and women with normal pregnancy. There were no significant differences between the two groups in the rate of nulliparity, rate of vaginal delivery, and intake of calories and protein from 30 weeks of pregnancy to 1 month postpartum.

### Changes in the muscle thicknesses of rectus femoris and vastus intermedius in women on bed rest during pregnancy

There were no significant changes in muscle thickness from 30 weeks of pregnancy to 1 month postpartum at all six sites in the longitudinal study.

### Comparison of the muscle thickness between women with normal pregnancy and women on bed rest during pregnancy

**Figure 2:**
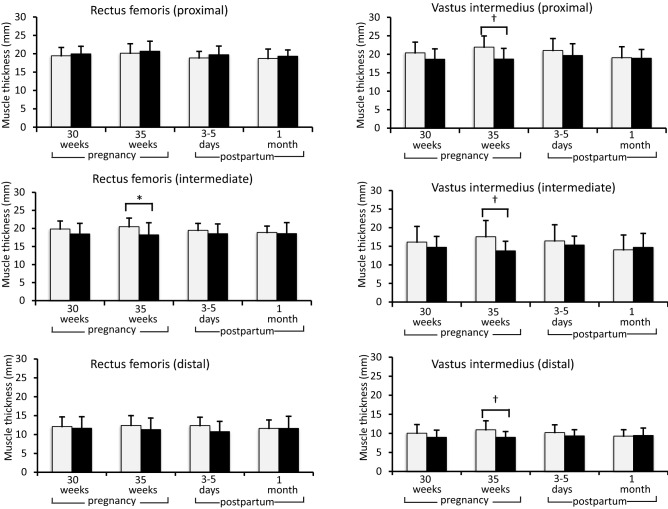
Comparison of muscle thickness changes between women with normal pregnancy and pregnant women on bed rest. Comparison of the changes in the muscle thickness of the rectus femoris (proximal, intermediate, and distal points) and vastus intermedius (proximal, intermediate, and distal points) between women with normal pregnancy (open bars) and women on bed rest during pregnancy (closed bars). Data are presented as mean ± standard deviation (*p < 0.05; ^†^p < 0.01).

At 30 weeks of pregnancy, there were no significant differences in the muscle thickness measurements at all six sites between women with normal pregnancy and women on bed rest. Muscle thicknesses measured at the proximal, intermediate, and distal points of the vastus intermedius and intermediate point of the rectus femoris at 35 weeks of pregnancy in women on bed rest were significantly (p < 0.01, p < 0.01, p < 0.01, and p < 0.05, respectively) lower than those in women with normal pregnancy (18.7 ± 3.0 vs. 21.9 ± 3.1 mm, 13.8 ± 2.7 vs. 17.6 ± 4.4 mm, 9.0 ± 1.6 vs. 10.9 ± 2.4 mm, and 18.2 ± 3.4 vs. 20.5 ± 2.4 mm, respectively). In the postpartum period, there were no significant differences in all muscle thicknesses between women on bed rest and women with normal pregnancy (Fig. 2).

### Associations between muscle thicknesses and duration of bed rest

The muscle thickness measurements at 30 weeks of pregnancy, those at 35 weeks of pregnancy, and the differences of muscle thickness between 30 and 35 weeks of pregnancy, were not significantly correlated with the duration of bed rest.

## Discussion

The current study has two significant findings. First, the quadriceps femoris muscle thickness in women with normal pregnancy increased gradually during pregnancy and showed a peak at 35 weeks of pregnancy followed by a decrease in the postpartum period. Second, in women on long-term bed rest for TPL, the quadriceps femoris muscle thickness showed no significant change during pregnancy and the postpartum period, resulting in a thinner quadriceps femoris muscle compared to women with normal pregnancy at 35 weeks of pregnancy. To the best of our knowledge, this is the first study to examine the longitudinal change in the muscle thickness in women during pregnancy and the postpartum period, and the influence of bed rest on muscle thickness as measured by ultrasonography.

Studies on non-pregnant women have reported that training increased the muscle thickness of the quadriceps femoris as measured by ultrasonography^18^, in addition to increasing the cross-sectional area of the lower limb muscles as measured by MRI^19^. However, to date, there have been no reports regarding the change in the muscle thickness and volume during pregnancy and the postpartum period. Using ultrasonography, we observed that the thickness of the quadriceps femoris muscle increased during pregnancy and decreased during puerperium. An increase in the thickness of the quadriceps, an antigravity muscle, may be induced by the increased load due to weight gain during pregnancy. Additionally, load reduction due to body weight loss after delivery may result in reduced muscle thickness of the quadriceps femoris. Meanwhile, maternal hormonal changes during pregnancy and the postpartum period may also affect the muscle thickness. Circulating estrogen levels increase toward the end of pregnancy and decrease during the postpartum period^20^. Estrogen is now considered to play a positive role in maintaining skeletal muscle function and volume because the declining estrogen level at least partially explains the accelerated muscle loss in postmenopausal women^21,22^. The mechanism by which estrogen effects muscle physiology is not clear yet. It has been reported that estrogen interferes with the expression of the inflammatory cytokine IL-6^23^. A higher IL-6 concentration in serum was reported to be associated with reduced muscle volume and strength^24^. Therefore, circulating estrogen levels may influence the increase in muscle thickness during pregnancy as well as the decrease in muscle thickness during puerperium.

Reduction in the gravity load on the quadriceps femoris muscle is known to cause muscle atrophy in men and non-pregnant women^7–9^. A previous ultrasonography study revealed that immobilization due to bed rest decreased the muscle thickness of the quadriceps femoris along with a loss in the muscle volume in non-pregnant women^7^. It has also been reported that in non-pregnant patients on bed rest in intensive care units, the quadriceps femoris thickness correlated negatively with length of hospital stay^9^. However, in the present study, women on bed rest treatment exhibited no changes in the quadriceps femoris muscle thickness during pregnancy and the postpartum period. Although we were unable to clarify the reason in the present study, we assume that the reduction in the gravity load on the quadriceps femoris muscle due to bed rest suppressed the increase in the muscle thickness, which was observed in women with normal pregnancy in the late third trimester. On the other hand, increased estrogen secretion during pregnancy may assist in sustaining muscle thickness and neutralizing the effect of bed rest on the muscle volume reduction.

We showed that a change in the muscle thickness in the intermediate part of the quadriceps femoris was more noticeable than that in the proximal and distal parts of the quadriceps femoris. A previous study on adult male healthy volunteers receiving bed rest revealed that the cross-sectional area (measured using MRI) of the belly part of the quadriceps femoris was lesser than the tendon part of the quadriceps femoris^25^. Similar results were also found in a study involving resistance training^26,27^. The influence of the gravity load may be different on the belly and tendon parts of the quadriceps femoris muscle.

In the present study, changes in the muscle thickness of the vastus intermedius were more marked than those in the rectus femoris. In a previous MRI study, it was observed that the rate of change in the cross-sectional area of the vastus intermedius was more marked than that in the rectus femoris in young adult men on bed rest only, compared to those performing resistance training during bed rest^26,27^. Anatomically, compared to the other three muscles of the quadriceps femoris, the vastus intermedius has a unique structure with the origin of the muscle fibers adhering directly to the femur, and easily transmitting muscle extension force to the femur^28^. Compared to the rectus femoris, the vastus intermedius is more likely to undergo muscle atrophy due to bed rest and muscle hypertrophy due to training.

The measurements obtained by integrating tomographic images from computed tomography and MRI are the gold standard for the evaluation of muscle volume^29^; however, their use in pregnant women is not favored because of the risk of radiation exposure, high cost, and lack of deployability. Muscle thickness evaluation by ultrasonography has been reported to correlate with the summation of the cross-sectional area of the muscle as measured by MRI^8^. It was also previously shown that muscle thickness of the rectus femoris and vastus intermedius measured by ultrasonography could indicate the muscle volume of the quadriceps femoris^8,30^. The use of ultrasonography is useful for evaluating muscle thickness in pregnant women because it provides a safe, portable, and non-invasive method. In the present study, we showed that ultrasonography was highly reproducible (test–retest reliability) as seen in previous studies in men and non-pregnant women^31,32^, and provided a practical method of evaluation of the muscle volume. Ultrasonographic evaluation of the quadriceps femoris muscle can be a useful tool for evaluating muscle thickness in pregnant women.

This study had several limitations. First, we treated pregnant women with TPL with ritodrine hydrochloride as well as restricted bed rest. Since ritodrine hydrochloride has been reported to cause rhabdomyolysis^33^, it may have affected the muscle thickness of the quadriceps femoris in this study. Further study is needed to clarify the effect of ritodrine hydrochloride on muscle volume. Second, we evaluated only the quadriceps femoris muscle, which is an antigravity muscle. We assumed that the increase in muscle thickness during normal pregnancy was caused by an increase in the load associated with the change in body weight during pregnancy, but other factors such as estrogen levels may also increase the muscle thickness, as stated above. Evaluation of the non-antigravity muscles, such as the biceps brachii, may be needed to clarify the reason for the change in muscle thickness during pregnancy. Third, although we measured the muscle thickness of the rectus femoris and vastus intermedius in line with previous studies to assess the muscle thickness of the quadriceps femoris muscle^34^, vastus lateralis or vastus medialis muscles may have different characteristics in pregnant women.

In conclusion, our study demonstrated that muscle thickness of the quadriceps femoris changed during pregnancy and the postpartum period, and the change was affected by prolonged bed rest. In women with normal pregnancy, muscle thickness of the quadriceps femoris increased during pregnancy and decreased in the postpartum period. Meanwhile, in pregnant women on bed rest treatment, the quadriceps femoris thickness showed no significant change during the pregnancy and postpartum periods, resulting in the muscle being significantly thinner in the late third trimester compared to that in women with normal pregnancy.

## Methods

### Study design and subjects

We performed two studies: a longitudinal study on the changes in the quadriceps femoris muscle thickness in women with normal pregnancy, both during pregnancy and the postpartum period (study 1), and a prospective comparison study on the quadriceps femoris muscle thickness during pregnancy and the postpartum period in women with normal pregnancy and women treated for TPL with bed rest (study 2) between June 2014 and March 2016. The Ethics Committee of Tokushima University Hospital reviewed and approved the study (Approval number 2037-1), and written informed consent was obtained from all participants. All experimental protocols were designed according to the Declaration of Helsinki’s ethical principles and performed in accordance with the Ethical Guidelines for Medical and Health Research Involving Human Subjects.

### Study 1

The study population consisted of 26 pregnant women aged 23–40 years. We recruited the participants from the outpatient clinic of the Department of Obstetrics and Gynecology, Tokushima University Hospital between June 2014 and March 2016 for the longitudinal study. Women with hypertensive disorders of pregnancy, diabetes mellitus, gestational diabetes mellitus, maternal complications requiring medication, prenatally diagnosed severe fetal malformations, and those who were prescribed bed rest for any other reason were excluded. All included women had singleton pregnancies lasting 37 weeks or more. Muscle thickness of the proximal, intermediate, and distal points of the rectus femoris and vastus intermedius muscles were measured using ultrasonography at 11–13, 26, 30, and 35 weeks of pregnancy, and at 3–5 days and 1 month postpartum. We used the food frequency questionnaire software (Excel Eiyoukun FFQg version 3.0; Kenpousha, Tokyo, Japan) for evaluating the caloric and protein intake of the outpatient participants^35^. Physical activity was assessed with the Japanese version of the international physical activity questionnaire (IPAQ), which was used to evaluate physical activity in different domains^36,37^. Each domain assesses walking, with moderate and vigorous physical activity performed for at least 10 min each day per week. By using the IPAQ, we calculated an average metabolic equivalents (METs) score for total physical activity performed per week in MET min/week^38^.

### Measurement of muscle thickness

**Figure 3:**
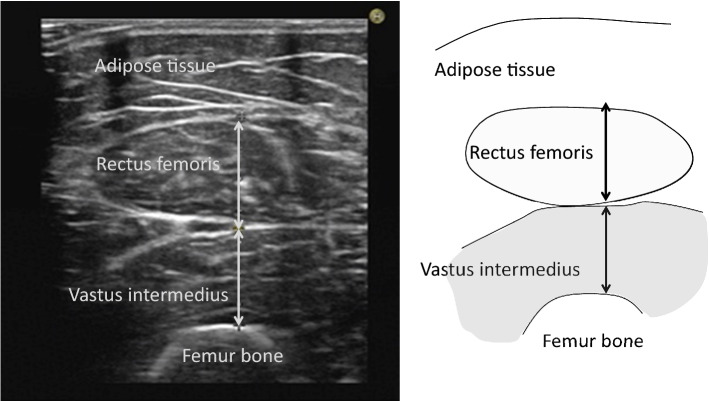
Ultrasound image of the rectus femoris and vastus intermedius.

The position of the participants during ultrasonographic measurements, and the choice of the site for measurements on limbs were the same as those described in a previous study^9^. We measured the muscle thicknesses of the quadriceps femoris at the anterior aspect of the right leg using a real-time B mode ultrasound imaging device (Hi Vision Preirus; Hitachi, Tokyo, Japan) with a 7.5 MHz linear-array probe (EUP-L74M). To improve acoustic coupling without compressing the dermal surface, a water-soluble transmission gel was placed over the scan head. The transducer was held perpendicular to the skin surface. Images were captured, stored on the hard disk of the ultrasound machine, and then muscle thickness was measured using on screen calipers (Fig. 3). All measurements were performed with the participants in the supine position, meaning full extension (passively) at the knee and elbow joints. Mean muscle thickness was calculated as the mean of five consecutive measurements of each muscle site, and all measurements were performed by the same experienced investigator.

### Sites for ultrasonic measurement

**Figure 4:**
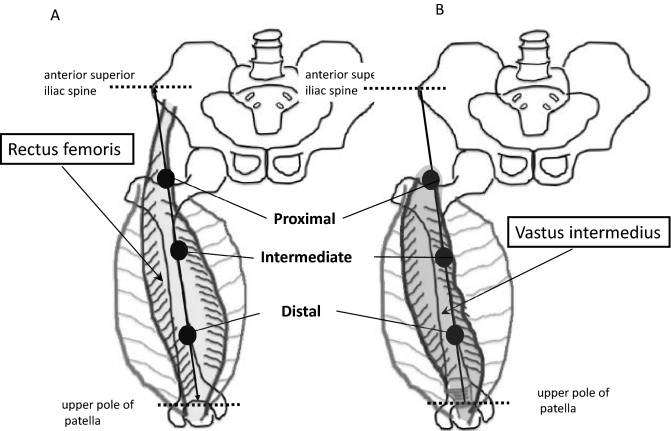
Ultrasonographic measurement sites on the rectus femoris (**A**) and vastus intermedius (**B**). Proximal point, on the anterior surface, 25% proximal to the point between the anterior superior iliac spine and upper pole of the patella; intermediate point, on the anterior surface midway between the anterior superior iliac spine and upper pole of the patella; distal point, on the anterior surface, 75% distal to the point between the anterior superior iliac spine and upper pole of the patella.

The quadriceps femoris muscle is subdivided into four separate muscles in front of the thigh: vastus lateralis, located in the lateral superficial part; vastus medialis, located in the medial superficial part; rectus femoris, located in the middle superficial part; and vastus intermedius, located between vastus laterals and vastus medialis, in the deep part of the front of the thigh. We measured the muscle thickness of the rectus femoris and vastus intermedius separately at the proximal, intermediate, and distal points. These points were determined as follows: proximal point, on the anterior surface, 25% proximal to the point between the anterior superior iliac spine and upper pole of the patella; intermediate point, on the anterior surface midway between the anterior superior iliac spine and upper pole of the patella; distal point, on the anterior surface, 75% distal to the point between the anterior superior iliac spine and upper pole of the patella (Fig. 4).

### Study 2

We recruited 15 pregnant women who were admitted to our hospital at less than 30 weeks of pregnancy and treated with bed rest for TPL. We compared the muscle thickness in these women with that in the 26 normal pregnant women included in study 1. Cases with regular objective uterine contractions and/or significant cervical changes (dilation and/or effacement and/or short cervical length) were diagnosed as TPL. All 15 women with TPL were treated with bed rest and ambulation restricted to bathroom privileges, and intravenous ritodrine hydrochloride. The bed rest and intravenous ritodrine hydrochloride were continued until 35–36 weeks of pregnancy. Dietary supplement in the hospital included 626 mg of calcium, 1145 mg of phosphorus, 7 μg of vitamin D, and 2000 kcal/day on an average. Women with hypertensive disorders of pregnancy, diabetes mellitus, gestational diabetes mellitus, maternal complications requiring medication, prenatally diagnosed severe fetal malformations and those who had received glucocorticoid treatment or magnesium sulfate treatment were excluded from the study. All women had singleton pregnancies lasting 35 weeks or more. We measured the muscle thickness at the same six sites as in study 1 at 30 and 35 weeks of pregnancy, and then at 3–5 days and 1 month postpartum. We used the same frequency questionnaire software as in study 1 for outpatient participants, and we calculated the intake of protein and calories from the hospital meals given to the inpatients. Physical activity was assessed using the same questionnaire as in study 1.

### Test–retest reliability of ultrasound measurements

To assess the reliability of the muscle thickness measurements, the observer made two sets of ultrasonic measurements on 17 pregnant and postpartum women who were not participating in study 1 and study 2. Two sets of measurements were taken 30 min apart. In each set, five measurements were performed at all six muscle sites. The investigator was blinded to the results of the measurements displayed on the ultrasound machine during the tests, and the order of the measurement sites was randomized in order to prevent memory bias. Intraclass correlation coefficients for the proximal point of the rectus femoris, intermediate point of the rectus femoris, distal point of the rectus femoris, proximal point of the vastus intermedius, intermediate point of the vastus intermedius, and distal point of the vastus intermedius were 0.95, 0.99, 0.99, 0.99, 0.99, and 0.99, respectively. Test–retest intra-examiner reliability was considered high for all measurements. Test–retest inter-examiner reliability was also high for all measurements, with the intraclass correlation coefficients for the proximal point of the rectus femoris, intermediate point of the rectus femoris, distal point of the rectus femoris, proximal point of the vastus intermedius, intermediate point of the vastus intermedius, and distal point of the vastus intermedius, being 0.77, 0.7, 0.75, 0.87, 0.99, and 0.84, respectively.

### Statistical analysis

Data were expressed as mean ± standard deviation, medians and IQRs, and proportions (%). Fisher’s exact test, student t-test, and Mann–Whitney U test were used for statistical analysis of the baseline characteristics. Differences in the muscle thickness between the groups were compared by the student’s t-test (in the cross-sectional study) or a repeated measures ANOVA (in the longitudinal study), while multiple comparisons in the longitudinal study were performed using the paired t-test with Bonferroni post-hoc correction. All statistical analyses were performed with EZR (Saitama Medical Center, Jichi Medical University, Saitama, Japan), which is a graphical user interface for R (R Foundation for Statistical Computing, Vienna, Austria). More specifically, it is a modified version of R commander designed to add the statistical functions that are frequently used in biostatistics^39^. All p-values were two-tailed, and α was set at a significance level of 0.05.

## Data Availability

The datasets analyzed during this study cannot be shared publicly as they contain sensitive patient information and are the property of Tokushima University Hospital. Consultation of the data by other interested researchers may be considered by Tokushima University Hospital on reasonable request to the corresponding author.
